# No Dye Fits All: Illuminating the Challenges of Fluorescent Extracellular Vesicle Labeling

**DOI:** 10.1002/jev2.70291

**Published:** 2026-05-05

**Authors:** Petra Vrdoljak, Alessandro Idrovo‐Gavilanes, Thu Huyen Nguyen, Amélie Vander Cruyssen, Niké Guilbert, Cláudio Pinheiro, Martina Fabiano, Martina Pannetta, Lien Lippens, An Hendrix

**Affiliations:** ^1^ Laboratory of Experimental Cancer Research Department of Human Structure and Repair Ghent University Ghent Belgium; ^2^ Cancer Research Institute Ghent Ghent Belgium; ^3^ Laboratory of Immunoregulation and Mucosal Immunology VIB‐UGent Center for Inflammation Research Ghent Belgium

**Keywords:** exosomes, microvesicles, nano‐flow cytometry, outer membrane vesicles, single‐particle analysis, super‐resolution microscopy, tracking

## Abstract

The widespread use of fluorescent dyes for extracellular vesicle (EV) labeling has substantially improved our ability to trace EVs *in vitro*, *ex vivo*, and *in vivo*. However, interpretability of these experiments remains limited by methodological challenges, including incomplete dye removal, dye‐associated artifacts, and intrinsic EV heterogeneity. In this study, we evaluated a range of mechanistically distinct fluorescent dyes, including lipophilic membrane‐ (Aco‐520, DiD, FM4‐64, PKH26) and protein‐associated dyes (CFSE, Cy5), across EVs of both prokaryotic and eukaryotic origin. EV labeling was assessed at the single‐vesicle level by nano‐flow cytometry, complemented by super‐resolution microscopy for visual confirmation. Our results demonstrated that EV labeling efficiency, fluorescence intensity, and background signal are not solely determined by dye chemistry but are also strongly influenced by EV biological origins. Furthermore, free dye removal and purification strategies markedly influenced nano‐flow cytometry readouts, underscoring the need to utilize and optimize approaches such as ultracentrifugation and size‐exclusion chromatography to effectively remove residual dye while minimizing EV loss and artifactual signals. Across all conditions, appropriate controls were critical to distinguish *bona fide* EV‐associated fluorescence from artifactual events. Overall, our findings reinforce that no single dye is universally suitable for EV labeling and underscore the necessity of a question‐driven, context‐aware experimental design. We therefore include a box with key questions to support informed decision‐making and improve rigor in EV labeling workflows. Addressing these guiding questions prior to experimentation, together with transparent reporting in platforms such as EV‐TRACK will improve reproducibility, data interpretation, and the robustness of EV research.

For more than a decade, fluorescent labeling has shaped extracellular vesicle (EV) biology by tracing biodistribution, illuminating uptake, and visualizing intercellular communication, yielding images that reinforce the notion that seeing is believing. However, the growing appreciation of EV heterogeneity has challenged the use of fluorescent labeling strategies (Hendrix et al. 2023). Fluorescent dyes represent an attractive alternative to fluorescently tagged antibody‐based approaches, combining broader labeling across diverse EV populations with simple protocols (Verweij et al. 2021). Yet, emerging evidence reveals that fluorescent dye‐based labeling is far from straightforward. Critical aspects of experimental design, including dye selection, labeling protocols, rigorous controls, effective dye‐removal procedures, and transparent reporting, are often overlooked (Van Deun et al. 2017). In this editorial, we highlight key considerations in fluorescent dye‐based EV labeling and how they may influence the interpretation of EV research, drawing on recent literature and illustrative observations.

Melling et al. (2022) found that fluorescent dyes (C5‐Maleimide‐Alexa633 and PKH26) label only a small subset of cell culture‐derived EVs. This limitation is expected to be more pronounced 1) in EVs derived from biofluids of increasing biological complexity, such as urine, blood, and feces; and 2) with diversity at the level of cell type (eukaryotic or prokaryotic), which differ substantially in membrane composition (De Langhe et al. 2024; Hendrix and De Wever 2022; Tulkens et al. 2020). To date, most labeling strategies used for prokaryotic EVs have been adapted from protocols originally developed for eukaryotic EVs (Rubio et al. 2020; Won et al. 2023). Under the same labeling conditions (**Figure** **S1A**), six widely used dyes (Aco‐520, CFSE, PKH26, Cy5, DiD, and FM4‐64; **Table** **1**) commonly applied to eukaryotic EVs, behaved differently when extended to EVs of eukaryotic and prokaryotic origin (**Figures** **1** **and** **2**). While these observations are intended as illustrative examples rather than a systematic comparison, they highlight that fluorescent dyes do not interact with all EVs equally, and their apparent success depends on the biological context in which they are used.

**TABLE 1:** Dye characteristics.

Dye	Product information	Catalog no. (supplier)	Type	Excitation/ emission (nm)
**Aco‐520**	Aco‐520	Aco‐520 (Acoerela)	Conjugated oligoelectrolyte dye that intercalates and partitions into lipid bilayers through electrostatic and hydrophobic interactions (Pham et al. 2023; Zhou et al. 2019)	488/520
**CFSE**	Vybrant CFDA‐SE	V12883 (Thermo Fisher)	Non‐fluorescent, cell‐permeant dye that is hydrolyzed by intracellular esterases to a fluorescent, amine‐reactive form, which covalently binds intracellular proteins (Loconte et al. 2023; Rubio et al. 2020; Woud et al. 2022)	492/517
**PKH26**	PKH26 Red Fluorescent Cell Linker Midi Kit for General Cell Membrane Labeling	MIDI26 (Sigma‐Aldrich)	Fluorescent dye with long aliphatic tails that integrate into lipid regions of the cell membrane (Haines et al. 2025; Pužar Dominkuš et al. 2018; Reclusa et al. 2020)	551/567
**Cy5**	Cy5 Mono NHS Ester	GEPA15104 (Cytiva)	Cyanine dye with a reactive NHS ester group that forms covalent amide bonds with primary amines on surface proteins (Kim et al. 2017; Won et al. 2023)	646/662
**DiD**	DiD' solid; DiIC18(5) solid (1,1'‐Dioctadecyl‐3,3,3',3'‐Tetramethylindodicarbocyanine, 4‐Chlorobenzenesulfonate Salt)	D7757 (Thermo Fisher)	Fluorescent dye with long‐chain dialkylcarbocyanines that insert into lipid bilayers (García‐Silva et al. 2021; Tripura et al. 2022)	644/665
**FM4‐64**	FM 4–64 Dye (N‐(3‐Triethylammoniumpropyl)‐4‐(6‐(4‐(Diethylamino) Phenyl) Hexatrienyl) Pyridinium Dibromide)	T13320 (Thermo Fisher)	Lipophilic styryl dye that inserts into the outer leaflet of lipid membranes (Lichius and Zeilinger 2019; Martínez‐Andrade et al. 2025)	515/640

**FIGURE 1:**
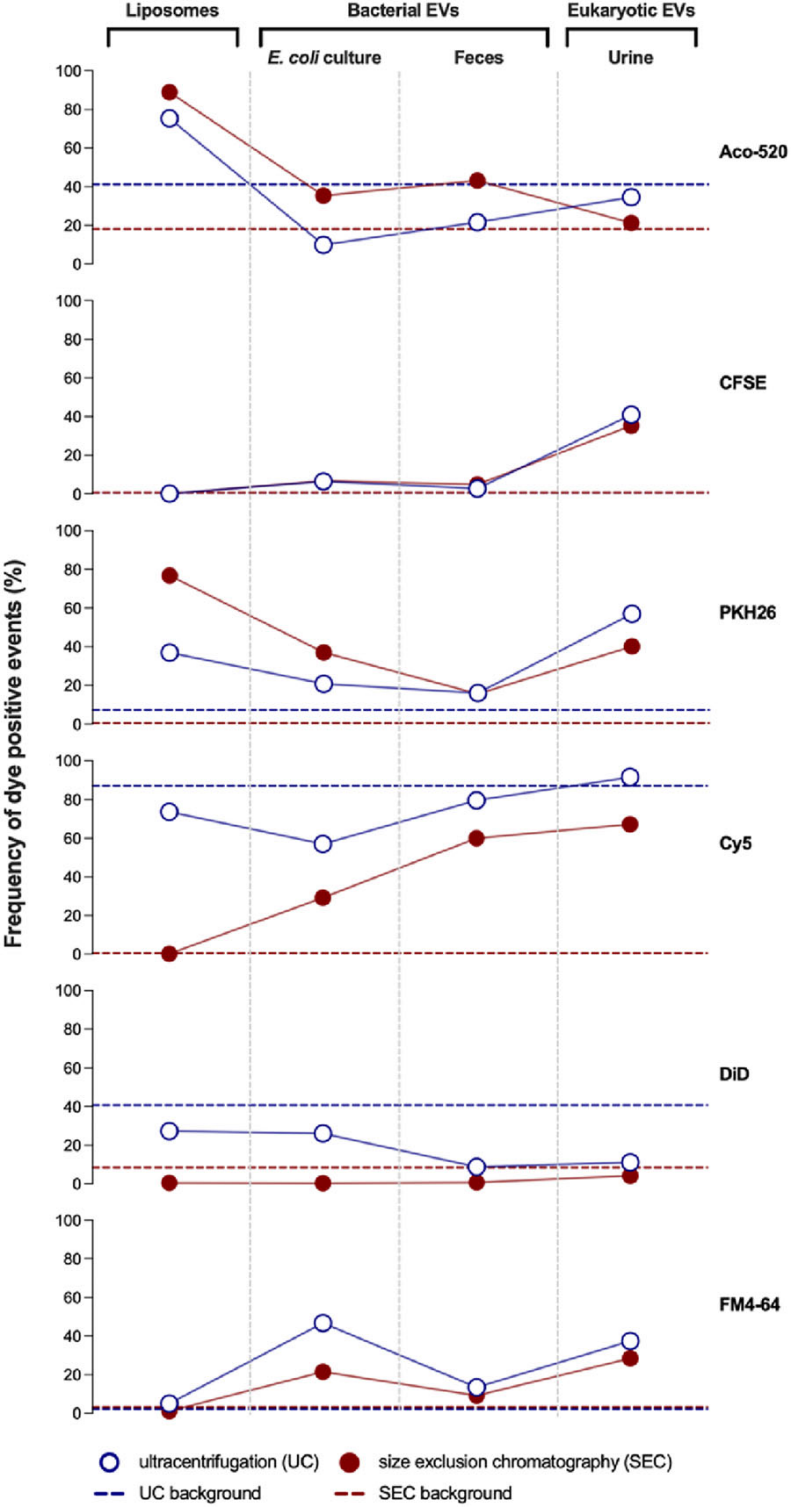
Frequency of dye positive events following unbound dye removal by ultracentrifugation (UC) and size‐exclusion chromatography (SEC). Percentages of dye positive single events from different nanoparticle samples (liposomes, *Escherichia coli* bacterial EVs, fecal bacterial EVs, and urinary eukaryotic EVs) labeled using six fluorescent dyes (Aco‐520, CFSE, PKH26, Cy5, DiD, and FM4‐64), including PBS (dashed lines) control for removal of the unbound dye by UC (blue) or SEC (red) as assessed by nano‐flow cytometry. Data shown represent a single measurement from pooled preparations (*n* = 1) and are presented solely as illustrative examples of dye‐dependent variability under one predefined labeling condition. Percentages reflect detectable fluorescent events within instrument thresholds and should not be interpreted as absolute measures of vesicle labeling efficiency.

**FIGURE 2:**
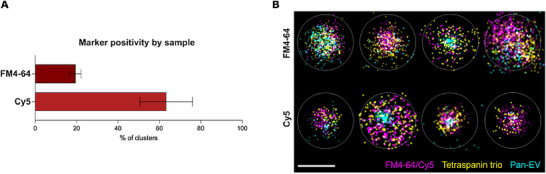
Super‐resolution microscopy visualization of FM4‐64 and Cy5‐labeled urinary eukaryotic EVs using the ONI nano‐imager. (A) Percentage of FM4‐64 and Cy5 labeled clusters and (B) representative imaged clusters of urinary eukaryotic EVs labeled using FM4‐64 and Cy5 with unbound dye removal by size‐exclusion chromatography (magenta), Tetraspanin trio‐561 (yellow) and Pan‐EV‐488 (cyan). Scale bar = 200 nm. Images and cluster proportions are presented as illustrative examples of dye‐dependent signal distribution and co‐localization patterns under a single predefined labeling condition.

The differences in context‐dependent fluorescent dye performance are further compounded by the tendency of dyes to generate signals that are unrelated to EVs altogether. Chen et al. (2023) demonstrated that dye‐related artifacts frequently dominated the detected signal when using PKH67, PKH26, di‐8‐ANEPPS, or Dil. Some dyes self‐associate into micelles or form fluorescent aggregates that may fall within the size range of EVs. Others bind non‐specifically to co‐isolated particles, such as lipoprotein particles in blood plasma, creating fluorescent structures that persist (Lozano‐Andrés et al. 2023). In line with these reports, dye‐only controls illustrated high background fluorescence across all evaluated dyes (**Figure** **3**), highlighting their necessity for identifying micelles, aggregates, and other non‐vesicular fluorescent particles. Furthermore, unstained EVs and buffer‐only controls establish baseline autofluorescence and instrument background signal, respectively. While additional controls, such as liposomes, allow for further validation of the dye's mechanism of action (**Figure** **S2A**).

**FIGURE 3:**
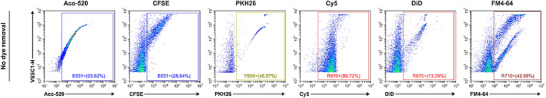
Dye‐only controls demonstrating background fluorescence. Nano‐flow cytometry plots showing PBS control samples labeled with six fluorescent dyes (Aco‐520, CFSE, PKH26, Cy5, DiD, and FM4‐64), without unbound dye removal. These data are presented to illustrate the potential contribution of dye‐derived fluorescent events, including aggregates or micelles.

Yet these controls alone are insufficient. Sterin et al. (2025) further demonstrated that the use of fluorescent dyes (DiD) requires dye removal strategies. Although the use of ultracentrifugation (UC), size‐exclusion chromatography (SEC), density gradient UC, filtration, or ion‐exchange approaches to remove unbound dye seems essential, it introduces additional challenges (Rautaniemi et al. 2022; Sterin et al. 2025). Dye aggregates may persist despite purification, producing scatter‐positive events that mimic EVs, as reflected in the markedly different performance of SEC and UC across the evaluated fluorescent dyes (**Figure** **1** and **Figure** **S2B**). At the same time, extensive post‐labeling purification may result in substantial particle loss, aggregation, or preferential retention of certain EV subpopulations. This reinforces that dye removal is not a single add‐on step but requires careful optimization of purification strategies that minimize dye‐derived artifacts while preserving EV yield and representativeness, as both may influence downstream biological interpretation.

Finally, Loconte et al. (2023) demonstrated that different fluorescent dyes (MemGlow‑488/640, CFSE, and DiO) can alter biological conclusions, using EV‐immune cell interactions to generate proof‐of‐concept. Therefore, beyond technical considerations, the choice of dye holds functional consequences: it can modify the narrative we construct about how EVs behave, with the risk that differences in labeling rather than differences in biology drive conclusions about EV‐cell communication. Some fluorescent dyes even transfer between membranes, producing false indications of EV uptake or cargo delivery, as reported by Takov et al. (2017).

Together, these illustrative observations, considered alongside recent literature, reinforce that fluorescent dye labeling is not a minor technical detail to be appended at the end of an experimental workflow, but a methodological choice that shapes every subsequent interpretation. The overall study context, entailing source origin, diversity in EV subsets, as well as the physicochemical characteristics of the dyes used, fundamentally complicates dye‐based labeling approaches. These pitfalls are well documented but remain inconsistently addressed in practice. Consequently, reliable fluorescent dye‐based EV labeling cannot be achieved through a uniform “add‐and‐wash” approach but instead requires context‐dependent strategies. To support robust and interpretable dye‐based EV studies, we propose a set of guiding questions (**Box** **1**) aimed at promoting thoughtful experimental design, careful optimization, and transparent reporting. Moving forward, progress in dye‐based EV research will depend not only on improved labeling reagents, but also on collective adherence to reproducibility practices, thorough validation, and consistent documentation through community‐driven resources such as MISEV, MIFlowCyt‐EV and EV‐TRACK (Lötvall et al. 2014; Théry et al. 2018; Van Deun et al. 2017; Welsh et al. 2024; Welsh et al. 2020). Ultimately, ensuring that fluorescent signals faithfully reflect EV biology will be essential to ensure that, in EV science, seeing is believing.

**Box 1. Guiding questions for EV labeling experimental design**

1. What is the study goal and EV context?
  - Determine the goal of the study (quantification, tracing biodistribution, tracking uptake, visualizing intracellular trafficking).
  - Specify sample types and expected EV heterogeneity (eukaryotic/prokaryotic, tissue/cell culture, biofluid, preparation method).
  - Identify any limitations that might affect labeling (low particle counts, non‐vesicular contaminants).
2. Which dye and labeling strategy best fit the study goal?
  - Choose a dye class (membrane intercalating, amine‐reactive, protein tags, etc.) that fits the goal and consider their physical properties (e.g., spectral profile, hydrophobicity/charge), reported EV performance, and any known artifact risks (e.g., lipophilic dyes are prone to form micelles).
  - If multiplexing, ensure minimal spectral overlap and compatible excitation/emission with available instrumentation.
3. Are essential controls included?
  All technical controls should be incorporated in accordance with the available guidelines (Welsh et al. 2020) and processed in an identical manner to EV‐containing samples. At a minimum, the following essential controls are included:
  - Buffer‐only control: measures instrument and procedural background.
  - Dye‐only control (no EVs): identifies unbound dye aggregates and fluorescent artifacts.
  - Unstained EVs: establish baseline autofluorescence and background from labeled samples.
  - Positive control EVs (when possible), which are well‐characterized and consistently labeled.
4. Have the labeling conditions been optimized?
  - Perform dye concentration titration using multiple concentrations that range from below to above manufacturer recommendations.
  - Optimize incubation time and temperature (e.g., room temperature or 37°C) that give the best signal‐to‐noise ratio.
5. How will unbound dye be removed and evaluated?
  - Compare purification methods (SEC, UC, density gradient, etc.) for dye removal efficiency and EV recovery.
  - Assess residual dye, EV yield, and size distribution using appropriate controls.
6. Are the experiments sufficiently reported to allow reproducibility?
  Document all experimental parameters and submit to community‐driven platforms, for example EV‐TRACK (https://www.evtrack.org/) (Van Deun et al. 2017) for transparency and reproducibility: dye identity and lot number, concentrations, incubation time/temperature, purification method, particle counts pre/post, instrument settings, and gating strategy.

## Author Contribution

**Petra Vrdoljak**: Conceptualization; Methodology; Data curation; Investigation; Visualization; Writing ‐ original draft; Writing ‐ review & editing. **Alessandro Idrovo Gavilanes**: Conceptualization; Methodology; Data curation; Investigation; Writing ‐ original draft; Writing ‐ review & editing. **Thu Huyen Nguyen**: Conceptualization; Methodology; Data curation; Investigation; Writing ‐ original draft; Writing ‐ review & editing. **Amélie Vander Cruyssen**: Conceptualization; Methodology; Investigation; Writing ‐ original draft. **Niké Guilbert**: Conceptualization; Methodology; Investigation; Writing ‐ original draft; Writing ‐ review & editing. **Cláudio Pinheiro**: Conceptualization; Methodology; Investigation; Writing ‐ original draft; Writing ‐ review & editing. **Martina Fabiano**: Writing ‐ review & editing. **Martina Pannetta**: Conceptualization; Methodology; Investigation; Writing ‐ review & editing. **Lien Lippens**: Conceptualization; Methodology; Investigation; Writing ‐ review & editing. **An Hendrix**: Conceptualization; Supervision; Funding acquisition; Writing ‐ review & editing.

## Conflicts of Interest

The authors declare that they have no competing interests.

## Supporting information



Supporting Information: jev270291‐sup‐0001‐SuppMatt.docx

## Data Availability

The data that support the findings of this study are available from the corresponding author upon reasonable request.
