# Supplementary material for: No Dye Fits All: Illuminating the Challenges of Fluorescent Extracellular Vesicle Labeling
Source: J Extracell Vesicles. 2026 May 5;15(5):e70291. doi: 10.1002/jev2.70291 (PMC13145352; doi:10.1002/jev2.70291)
Supplement: Supplementary file 1 — Supporting Information: jev270291‐sup‐0001‐SuppMatt.docx [file JEV2-15-e70291-s001.docx]

**SUPPLEMENTARY INFORMATION**

**Title:**

No dye fits all: illuminating the challenges of fluorescent extracellular vesicle labeling

**Authors:**

Petra Vrdoljak^1,2,3^*, Alessandro Idrovo Gavilanes^1,2^*, Thu Huyen Nguyen^1,2^*, Amélie Vander Cruyssen^1,2^*, Niké Guilbert^1,2^*, Cláudio Pinheiro^1,2^, Martina Fabiano^1,2^, Martina Pannetta^1,2^, Lien Lippens^1,2^, An Hendrix^1,2#^

**Affiliations:**

^1^ Laboratory of Experimental Cancer Research, Department of Human Structure and Repair, Ghent University, Ghent, Belgium

^2^ Cancer Research Institute Ghent, Ghent, Belgium

^3^ Laboratory of Immunoregulation and Mucosal Immunology, VIB-UGent Center for Inflammation Research, Ghent University, Ghent, Belgium

^#^ Corresponding author: [an.hendrix@ugent.be](mailto:an.hendrix@ugent.be)

* Equal contribution

**Keywords:**

# exosomes, microvesicles, outer membrane vesicles, tracking, nano-flow cytometry, super-resolution microscopy, single-particle analysisMaterials and methods

The experimental procedures described below were performed to generate illustrative examples supporting the methodological discussion. They were not designed as a comprehensive or optimized comparative study.

## Ethical approval

This study involved the collection and processing of fecal and urinary samples from healthy adult volunteers. Approval was granted by the Ethical Committee of Ghent University Hospital (project EC UZG 2014/0655). Written informed consent was obtained from all participants before sample donation.

## EV preparation from urine, feces and bacterial culture supernatant

Urinary extracellular vesicles (uEVs) were prepared and characterized from urine samples following the protocol described by Dhondt et al. (2020). Bacterial extracellular vesicles (BEVs) from *Escherichia coli* Nissle 1917 (EcBEVs) were prepared from bacterial cultures, while fecal BEVs (fBEVs) were prepared from human fecal samples. Both EcBEVs and fBEVs were prepared and characterized according to the protocol outlined by Tulkens et al. (2020).

## Liposomes

Phosphatidylserine and phosphatidylcholine (DOPS:DOPC, 50:50 molar ratio, 10 mM) containing anionic liposomes were used as a non-EV control (cat. no. CPS-505, Sigma-Aldrich).

## Nanoparticle labeling

Six different dyes were tested for exogenous nanoparticle labeling (**Table 1**). Each dye was reconstituted and diluted to a final working concentration of 25 µM. For all labeling conditions, an equal particle concentration of 5 × 10^10^ particles/mL (measured previously by CytoFLEX nano-flow cytometer) and 5 µM of dye were used. Samples were incubated for 30 min at 37 °C at 500 rpm. Dye-only controls (dye only in phosphate-buffered saline (PBS, pH 7.2, cat no. 20012019, Thermo Fisher), without EVs) were included to evaluate background fluorescence and potential dye aggregation.

After incubation, samples were processed under three different conditions. For the no dye removal condition, samples were diluted in PBS and directly analyzed using the CytoFLEX nano-flow cytometer. For ultracentrifugation, samples were diluted in 16 mL PBS and subjected to ultracentrifugation at 100,000 × g for 70 min at 4 °C. For size-exclusion chromatography (SEC), samples were diluted in 2 mL PBS and loaded onto a 10 mL SEC column (CL-2B beads packed in a 10 mL syringe); fractions 4 – 7 were collected, pooled, and concentrated to a final volume of 1 mL. An overview of the experimental setup is provided in **Supplementary Figure 1A**.

## Nano-flow cytometry

Labeled nanoparticles (EVs and liposomes) were analyzed using the CytoFLEX nano-flow cytometer (Beckman Coulter). Daily instrument quality control was performed according to manufacturer protocols. For each condition, 150,000 events were acquired at a flow rate of 1 µL/min and acquisition speed of 2000 – 5000 events/s to minimize the risk of swarming. Labeling efficiency was determined as the proportion of dye-positive particles, excluding debris and doublets (**Supplementary Figure 1B**). Particle (event) concentration (events/mL) was quantified using the CytoFLEX platform, which provided an additional readout.

## Super-resolution microscopy

Super-resolution images of Cy5 and FM4-64-labeled uEVs (with unbound dye removal using SEC) were acquired using direct stochastic optical reconstruction microscopy, dSTORM [Nano-imager S, Oxford Nanoimaging (ONI), UK], equipped with a 100X, 1.4NA oil immersion objective. The EV Profiler Kit 2 (Oxford Nanoimaging) was used per manufacturer's instructions. Surface marker analysis of EV samples was performed using Pan-EV-488 and Tetratspanin trio-561 antibodies supplied in the kit. Image acquisition was performed in total internal reflection fluorescence (TIRF) mode with a 52-53° angle at a set temperature of 32°C. Laser excitation was performed with laser powers set to 50% for 488 nm and 561 nm channels, and 30% for the 640 nm channel. Calibration was carried out prior to imaging using TeTraSpek microsphere (#T7279, Thermo Fisher Scientific) to ensure channel alignment within a 10 nm standard deviation. A total of 1000 frames of data were acquired (500 per channel) with a 30 ms exposure using AutoEV in CODI software (<https://alto.codi.bio/>, Oxford Nanoimaging). Localization images were drift-corrected, filtered by photon count and localization precision, and subsequently clustered and quantified to obtain population statistics and relative fluorophore abundance.

# Supplementary figures


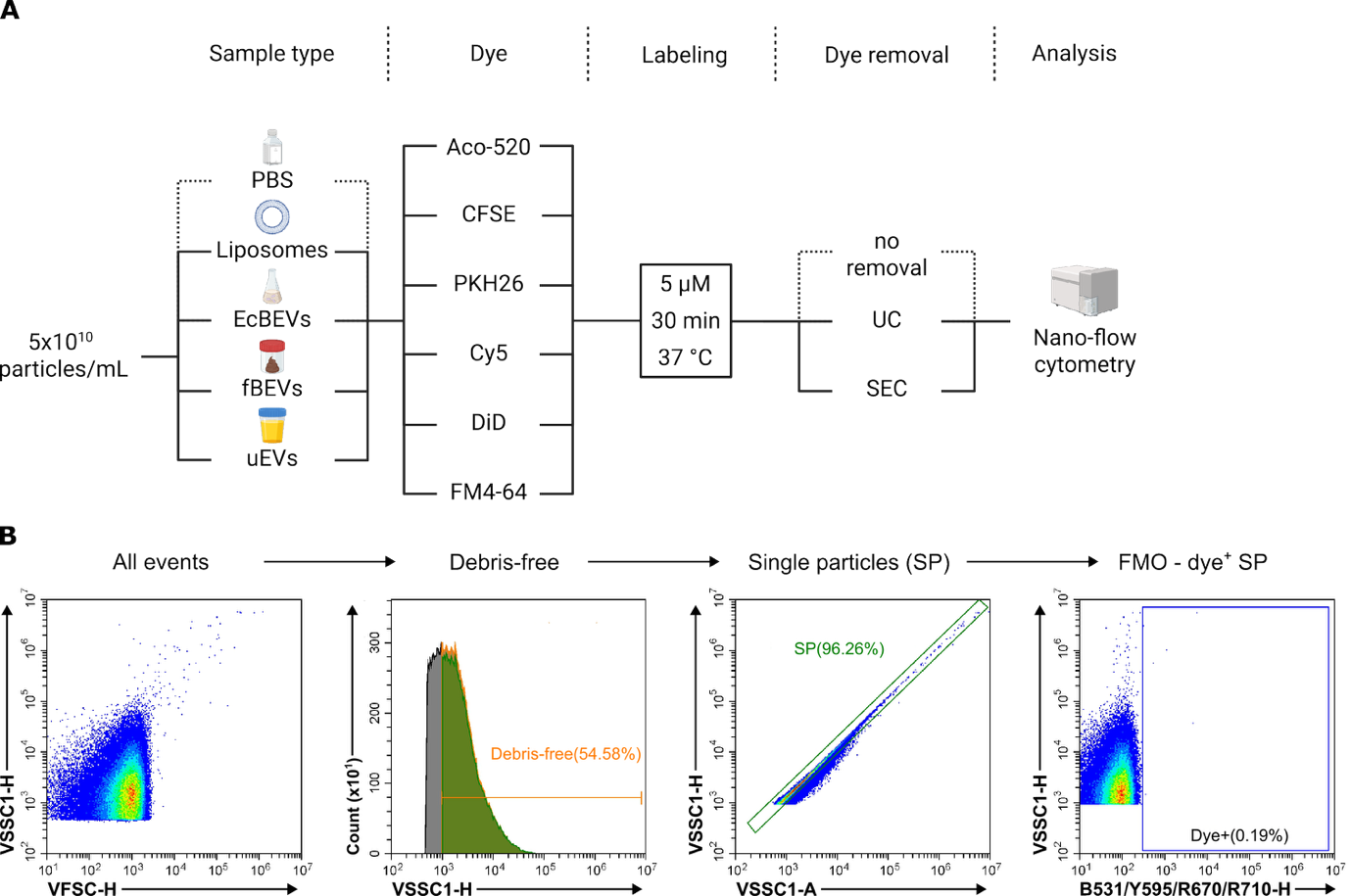


**Supplementary figure 1: Experimental set-up.** **(A)** Schematic overview of the experimental set-up. Briefly, different nanoparticles – liposomes, *Escherichia coli* bacterial EVs (EcBEVs), fecal bacterial EVs (fBEVs), and urinary eukaryotic EVs (uEVs) – or PBS (negative control) were labeled with six fluorescent dyes, followed by removal of unbound dye using ultracentrifugation (UC) or size-exclusion chromatography (SEC). Samples were then analyzed by nano-flow cytometry. **(B)** Representative gating strategy used for the assessment of dye-positive single particles using nano-flow cytometry.


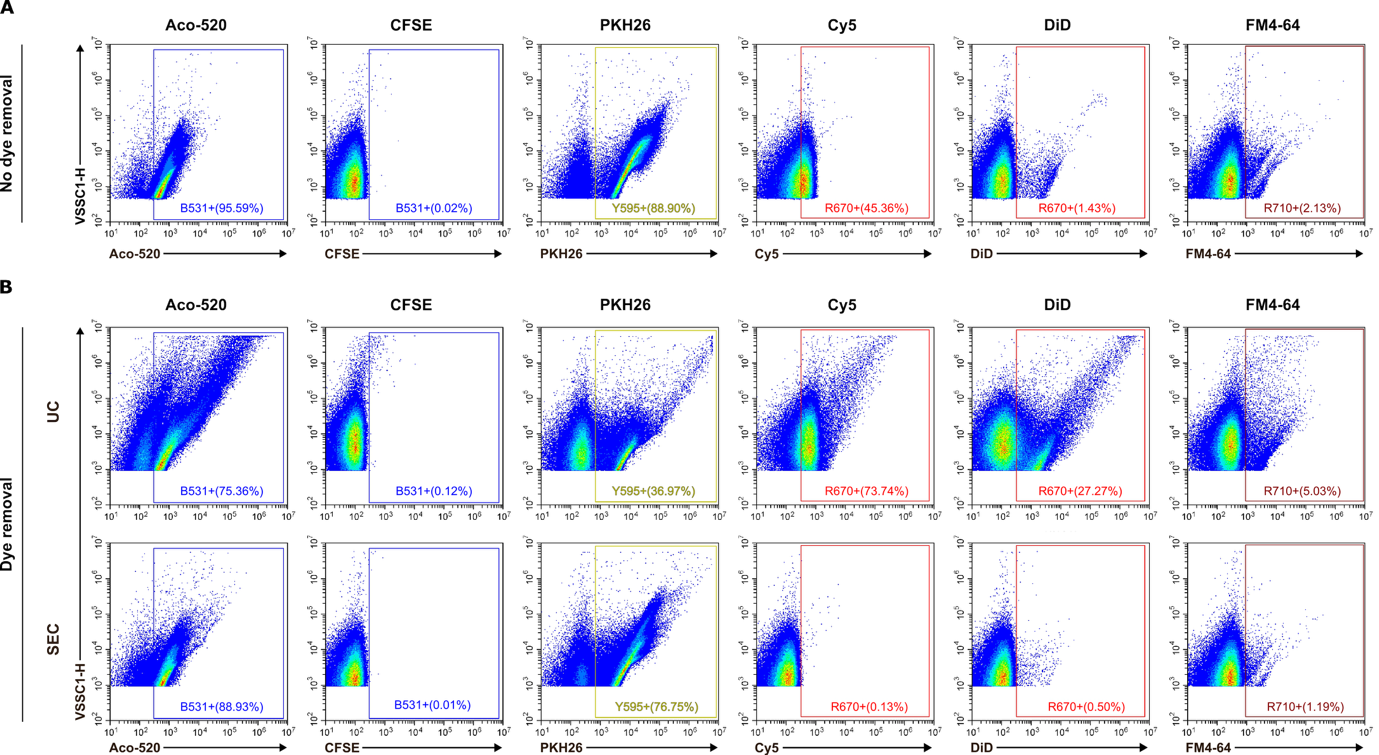


**Supplementary figure 2:** Nano-flow cytometry plots illustrating fluorescent signal intensity for liposomes labeled with six dyes (Aco-520, CFSE, PKH26, Cy5, DiD, and FM4-64). **(A)** No unbound dye removal, and **(B)** unbound dye removal using ultracentrifugation (UC) and size-exclusion chromatography (SEC). Data are presented as illustrative examples of dye-dependent signal behavior and potential background contribution.

# References

Dhondt, B., Lumen, N., De Wever, O., & Hendrix, A. (2020). Preparation of Multi-omics Grade Extracellular Vesicles by Density-Based Fractionation of Urine. *STAR Protoc*, *1*(2), 100073. <https://doi.org/10.1016/j.xpro.2020.100073>

Tulkens, J., De Wever, O., & Hendrix, A. (2020). Analyzing bacterial extracellular vesicles in human body fluids by orthogonal biophysical separation and biochemical characterization. *Nature Protocols*, *15*(1), 40–67. <https://doi.org/10.1038/s41596-019-0236-5>
